# Correction: Exploring the effect of menstrual loss and dietary habits on iron deficiency in teenagers: A cross-sectional study

**DOI:** 10.1371/journal.pone.0343133

**Published:** 2026-02-17

**Authors:** Lisa Söderman, Anna Stubbendorff, Linnea V. Ladfors, Beata Borgström Bolmsjö, Peter Nymberg, Moa Wolff

In S1 File, S1 Fig, S1 Table–S5 Table, the attached files do not match the headings. Please view the correct S1 File, S1 Fig, S1 Table - S5 Table below.

## Supporting information

S1 File Questionnaire. (DOCX)

S1 Fig Directed acyclic graph for identifying confounders between HMB and ID. (DOCX)

S1 Table General health in the study population, based on heavy menstrual bleeding. (DOCX)

S2 Table The use of iron supplements by menstrual status and dietary status, respectively. (DOCX)

S3 Table Levels of ferritin and hemoglobin, and rates of anemia and iron deficiency with cutoffs at ferritin<15 µg and <30 µg, respectively. Data are presented overall and by dietary status and heavy menstrual bleeding (HMB).
(DOCX)

S4 Table Logistic regression analysis, crude and adjusted for BMI and years of menstruation, showing the odds ratio (OR) with 95% confidence interval (CI) for serum ferritin <30 µg/L. (DOCX)

S5 Table Rates of anemia (hemoglobin < 120g/L) among participants depending on heavy menstrual bleeding (HMB) status, BMI group and dietary preference. χ^2^ test or Fisher’s exact test. Row percentages.
(DOCX)
