# Supplementary material for: Exploring the effect of menstrual loss and dietary habits on iron deficiency in teenagers: A cross-sectional study
Source: PLoS One. 2025 Dec 3;20(12):e0336688. doi: 10.1371/journal.pone.0336688 (PMC12674527; doi:10.1371/journal.pone.0336688)
Supplement: S1 File — (DOCX) [file pone.0336688.s001.docx]

Exploring the effect of menstrual loss and dietary habits on iron deficiency in teenagers: a cross-sectional study


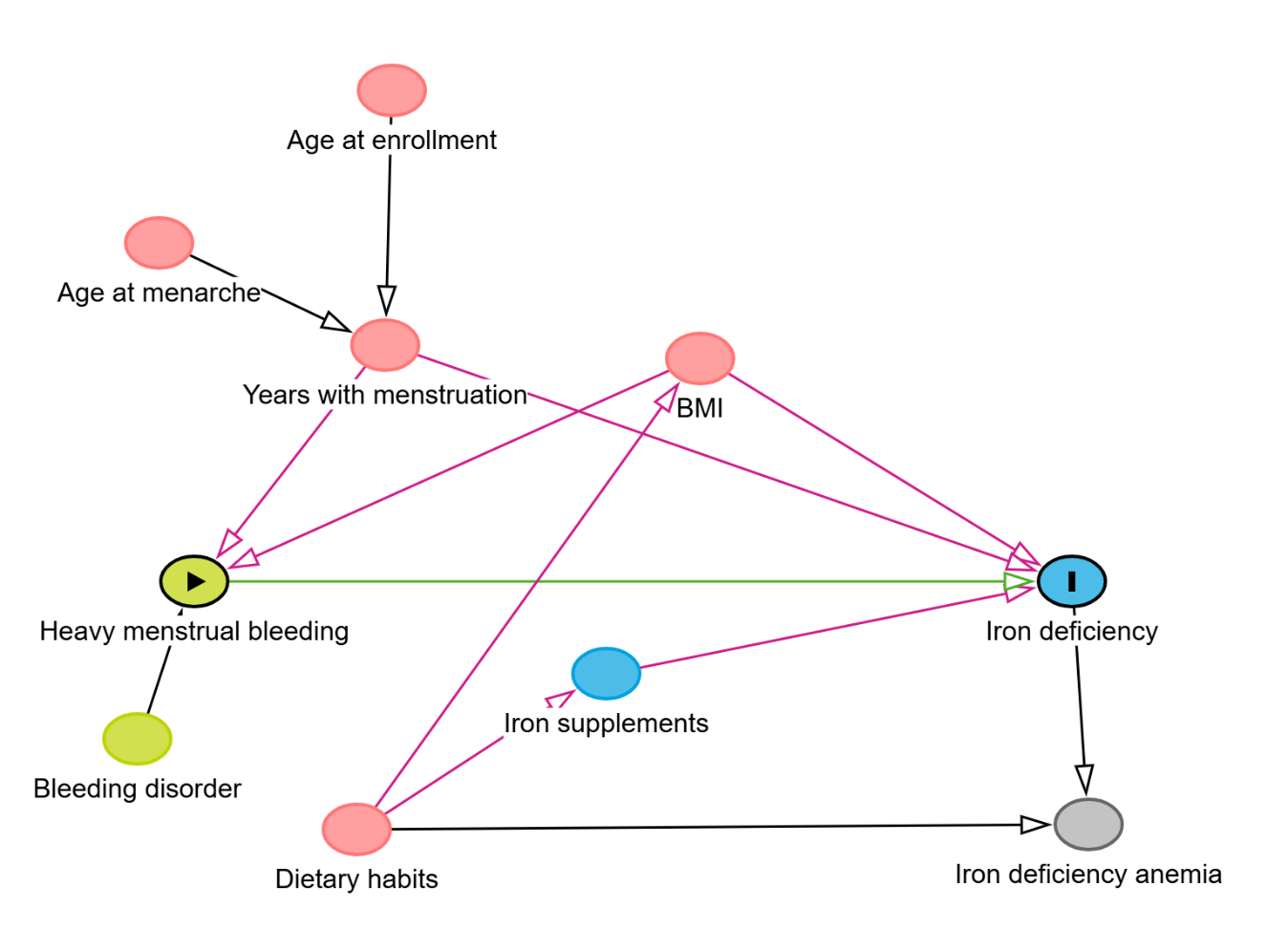


S1 Figure. Directed acyclic graph for identifying confounders between heavy menstrual bleeding (HMB) and iron deficiency (ID).
Green arrow indicates the “causal” relationship of interest, i.e. between HMB and ID. Red arrows indicate potential biasing paths. Black arrows indicate non-biasing, non-causal paths between variables. For estimating the total effect of HMB on ID, the Dagitty tool ([www.dagitty.net](http://www.dagitty.net)) suggests adjustment for BMI (body mass index) and years with menstruation.
