# Supplementary material for: Exploring the effect of menstrual loss and dietary habits on iron deficiency in teenagers: A cross-sectional study
Source: PLoS One. 2025 Dec 3;20(12):e0336688. doi: 10.1371/journal.pone.0336688 (PMC12674527; doi:10.1371/journal.pone.0336688)
Supplement: S1 Fig — (DOCX) [file pone.0336688.s002.docx]

Exploring the effect of menstrual loss and dietary habits on iron deficiency in teenagers: a cross-sectional study

S1 Table. General health in the study population, based on heavy menstrual bleeding (HMB) status. Row percentages.

|  | Good general health | Poor general health | *p*-value |
| --- | --- | --- | --- |
| Non HMB | 129 (69.4%) | 57 (30.6%) | 0.300 |
| HMB | 134 (64.4%) | 74 (35.6%) |  |
