# Supplementary material for: Exploring the effect of menstrual loss and dietary habits on iron deficiency in teenagers: A cross-sectional study
Source: PLoS One. 2025 Dec 3;20(12):e0336688. doi: 10.1371/journal.pone.0336688 (PMC12674527; doi:10.1371/journal.pone.0336688)
Supplement: S1 Table — (DOCX) [file pone.0336688.s003.docx]

Exploring the effect of menstrual loss and dietary habits on iron deficiency in teenagers: a cross-sectional study

S2 Table. The use of iron supplements by menstrual status and dietary status, respectively. Row percentages.

|  | | Iron supplement | | | |
| --- | --- | --- | --- | --- | --- |
|  | | Yes | No | | *p*-value |
| HMB | | 19 (7.5%) | 236 (92.5%) | | 0.930 |
| No HMB | | 16 (7.2%) | 205 (92.8%) | |  |
|  | |  |  | |  |
| Meat-restricted diet | | 15 (11.6%) | 114 (88.4%) | | 0.024 |
| Omnivore | | 20 (5.6%) | 335 (94.4%) | |  |
|  | |  |  | |  |
| Omnivore | | 20 (5.6%) | 335 (94.4%) | | 0.024 |
| No red meat | | 1 (3.7%) | 26 (96.3%) | |  |
| Pescetarian | | 4 (10.3%) | 35 (89.7%) | |  |
| Vegan/Vegetarian | | 10 (15.9%) | 53 (84.1%) | |  |
|  | |  |  | |  |
| Hemoglobin<120 g/L | | 3 (8.1%) | 34 (91.9%) | | 0.83 |
| Hemoglobin ≥120 g/L | 32 (7.2%) | | 415 (92.8%) |  | |
