# Supplementary material for: Exploring the effect of menstrual loss and dietary habits on iron deficiency in teenagers: A cross-sectional study
Source: PLoS One. 2025 Dec 3;20(12):e0336688. doi: 10.1371/journal.pone.0336688 (PMC12674527; doi:10.1371/journal.pone.0336688)
Supplement: S2 Table — (DOCX) [file pone.0336688.s004.docx]

Exploring the effect of menstrual loss and dietary habits on iron deficiency in teenagers: a cross-sectional study

S3 Table. Levels of ferritin and hemoglobin, and rates of anemia and iron deficiency with cutoffs at ferritin<15 µg and <30 µg respectively. Data presented overall and by dietary status and heavy menstrual bleeding (HMB). Row percentages.

|  | | Ferritin, median (IQR) | *p*-value | Ferritin  <15µg/L | Ferritin ≥15µg/L | *p*-value | Ferritin <30µg/L | Ferritin ≥30µg/L | *p*-value | Hemoglobin  (median (IQR)) | *p*-value | Hemoglobin <120 g/L | Hemoglobin ≥120 g/L | *p*-value |
| --- | --- | --- | --- | --- | --- | --- | --- | --- | --- | --- | --- | --- | --- | --- |
| All n=394 | | 18.0 (10.0-30.3) |  | 157 (39.8%) | 237 (60.2%) |  | 290 (73.6%) | 104 (26.4%) |  | 132.0 (126.0-139.0) |  | 34 (8.6%) | 360 (91.4%) |  |
| HMB (n=208; 52.8%) | | 14.0 (8.0-25.8) | <0.001 | 108 (51.9%) | 100 (48.1%) | <0.001 | 166 (79.8%) | 42 (20.2%) | 0.003 | 131.0 (124.0-138.0) | 0.002 | 26 (12.5%) | 182 (87.5%) | 0.004 |
| No HMB (n=186; 47.2%) | | 22.0 (14.0-37.0) |  | 49 (26.3%) | 137 (73.7%) |  | 124 (66.7%) | 62 (33.3%) |  | 134.0 (127.0-139.0) |  | 8 (4.3%) | 178 (95.7%) |  |
| Meat-restricted diet (n=110; 27.9%) | | 12.0 (7.8-22.0) | <0.001 | 68 (61.8%) | 42 (38.2%) | <0.001 | 92 (83.6%) | 18 (16.4%) | 0.005 | 129.5 (124.0-136.3) | 0.005 | 12 (10.9%) | 98 (89.1%) | 0.316 |
| Omnivore (n=284; 72.1%) | | 21.0 (12.0-32.0) |  | 89 (31.3%) | 195 (68.7%) |  | 198 (69.7%) | 86 (30.3%) |  | 134.0 (127.0-139.0) |  | 22 (7.7%) | 262 (92.3%) |  |
| Meat-restricted diet | HMB (n=55; 14.0%) | 11.0 (5.0-19.0) | <0.001 | 39 (70.9%) | 16 (29.1%) | <0.001 | 46 (83.6%) | 9 (16.4%) | <0.001 | 127.0 (122.0-135.0) | <0.001 | 8 (14.5%) | 47 (85.5%) | 0.013 |
|  | No HMB (n=55; 14.0%) | 13.0 (9.0-22.0) |  | 29 (52.7%) | 26 (47.3%) |  | 46 (83.6%) | 9 (16.4%) |  | 132.0 (125.0-137.0) |  | 4 (7.3%) | 51 (92.7%) |  |
| Omnivore | HMB (n=153; 38.8%) | 16.0 (9.0-27.0) |  | 69 (45.1%) | 84 (54.9%) |  | 120 (78.4%) | 33 (21.6%) |  | 132.0 (125.0-139.0) |  | 18 (11.8%) | 135 (88.2%) |  |
|  | No HMB (n=131; 33.2%) | 26.0 (16.0-40.0) |  | 20 (15.3%) | 111 (84.7%) |  | 78 (59.5%) | 53 (40.5%) |  | 135.0 (128.0-139.0) |  | 4 (3.1%) | 127 (96.9%) |  |
