# Supplementary material for: Exploring the effect of menstrual loss and dietary habits on iron deficiency in teenagers: A cross-sectional study
Source: PLoS One. 2025 Dec 3;20(12):e0336688. doi: 10.1371/journal.pone.0336688 (PMC12674527; doi:10.1371/journal.pone.0336688)
Supplement: S3 Table — Data are presented overall and by dietary status and heavy menstrual bleeding (HMB). (DOCX) [file pone.0336688.s005.docx]

Exploring the effect of menstrual loss and dietary habits on iron deficiency in teenagers: a cross-sectional study

S4 Table. Logistic regression analysis, crude and adjusted for BMI and years of menstruation, showing the odds ratio (OR) with 95% confidence interval (CI) for serum ferritin <30 µg/L.

|  | OR [95% CI] | aOR [95% CI] |
| --- | --- | --- |
| Meat-restricted diet and non HMB | 3.5 [1.7, 7.7] | 3.4 [1.5, 7.5] |
| Omnivore and HMB | 2.5 [1.7, 4.2] | 2.5 [1.5, 4.3] |
| Meat-restricted diet and HMB | 3.5 [1.6, 7.7] | 3.6 [1.6, 8.1] |
| Omnivore and non HMB | Ref | Ref |
| Meat-restricted diet | 2.2 [1.3, 3.9] | 2.2 [1.3, 3.9] |
| Omnivore | Ref | Ref |
| HMB | 2.0 [1.3, 3.1] | 2.0 [1.3, 3.2] |
| Non HMB | Ref | Ref |
| Takes iron supplement | 0.6 [0.3, 1.4] | 0.6 [0.3, 1.3] |
| No iron supplement | Ref | Ref |
| Years of menstruation | 1.0 [0.8, 1.2] |  |
| BMI | 0.9 [0.9, 1.0] |  |
