# Supplementary material for: Exploring the effect of menstrual loss and dietary habits on iron deficiency in teenagers: A cross-sectional study
Source: PLoS One. 2025 Dec 3;20(12):e0336688. doi: 10.1371/journal.pone.0336688 (PMC12674527; doi:10.1371/journal.pone.0336688)
Supplement: S4 Table — (DOCX) [file pone.0336688.s006.docx]

Exploring the effect of menstrual loss and dietary habits on iron deficiency in teenagers: a cross-sectional study

S5 Table. Rates of anemia (hemoglobin <120g/L) among participants depending on heavy menstrual bleeding (HMB) status, BMI group, and dietary preference. χ2 test or Fisher’s exact test. Row percentages.

|  | Hemoglobin <120 g/L  n=34 | Hemoglobin ≥120 g/L  n=360 | *p*-value |
| --- | --- | --- | --- |
| HMB | 26 (12.5%) | 182 (87.5%) | 0.004 |
| Non- HMB | 8 (4.3%) | 178 (95.7%) |  |
| BMI <18.5 | 7 (18.4%) | 31 (81.6%) | 0.042 |
| BMI 18.5-24 | 20 (7.9%) | 232 (92.1%) |  |
| BMI >25 | 1 (2.6%) | 37 (97.4%) |  |
| Meat-restricted diet | 22 (7.7%) | 262 (92.3%) | 0.316 |
| Omnivore | 12 (10.9%) | 98 (89.1%) |  |
