# Supplementary material for: Exploring the effect of menstrual loss and dietary habits on iron deficiency in teenagers: A cross-sectional study
Source: PLoS One. 2025 Dec 3;20(12):e0336688. doi: 10.1371/journal.pone.0336688 (PMC12674527; doi:10.1371/journal.pone.0336688)
Supplement: S5 Table — (DOCX) [file pone.0336688.s007.docx]

Exploring the effect of menstrual loss and dietary habits on iron deficiency in teenagers: a cross-sectional study

S6 Table. Sensitivity analysis, excluding cases with imputed data. N=328 complete case analysis. Logistic regression analysis showing crude (odds ratio) OR and 95% confidence interval (CI) for ID (iron deficiency) and adjusted OR for years of menstruation and BMI.

|  | OR | aOR |
| --- | --- | --- |
| Meat-restricted diet and HMB | 14.8 [6.5, 33.6] | 14.8 [6.5, 33.8] |
| Meat-restricted diet and non HMB | 8.2 [3.7, 18.7] | 8.0 [3.6, 17.8] |
| Omnivore and HMB | 4.1 [2.2, 7.6] | 4.1 [2.2, 7.8] |
| Omnivore and non HMB | Ref | Ref |
| Meat-restricted diet | 4.7 [2.8, 7.9] | 4.6 [2.8, 7.8] |
| Omnivore | Ref | Ref |
| HMB | 2.7 [1.7, 4.3] | 2.7 [1.7, 4.4] |
| Non HMB | Ref | Ref |
| Takes iron supplement | 0.3 [0.1, 1.1] | 0.3 [0.1, 1.0] |
| No iron supplement | Ref | Ref |
| Years of menstruation | 1.0 [0.9, 1.2] | - |
| BMI | 1.0 [0.9, .1.1] | - |
