# Supplementary material for: Exploring the effect of menstrual loss and dietary habits on iron deficiency in teenagers: A cross-sectional study
Source: PLoS One. 2025 Dec 3;20(12):e0336688. doi: 10.1371/journal.pone.0336688 (PMC12674527; doi:10.1371/journal.pone.0336688)
Supplement: Attachment — Submitted filename: Review kommentarer och svar PLOSonev15 Oct 11.docx [file pone.0336688.s010.docx]

PONE-D-25-27936

Iron insight - Exploring the effect of menstrual loss and dietary iron intake on iron deficiency in teenagers: a cross-sectional study

PLOS ONE

Dear Dr. Söderman,

Thank you for submitting your manuscript to PLOS ONE. After careful consideration, we feel that it has merit but does not fully meet PLOS ONE’s publication criteria as it currently stands. Therefore, we invite you to submit a revised version of the manuscript that addresses the points raised during the review process.

Please submit your revised manuscript by Oct 09 2025 11:59PM. If you will need more time than this to complete your revisions, please reply to this message or contact the journal office at [plosone@plos.org](mailto:plosone@plos.org). When you're ready to submit your revision, log on to <https://www.editorialmanager.com/pone/> and select the 'Submissions Needing Revision' folder to locate your manuscript file.

Please include the following items when submitting your revised manuscript:

- A rebuttal letter that responds to each point raised by the academic editor and reviewer(s). You should upload this letter as a separate file labeled 'Response to Reviewers'.
- A marked-up copy of your manuscript that highlights changes made to the original version. You should upload this as a separate file labeled 'Revised Manuscript with Track Changes'.
- An unmarked version of your revised paper without tracked changes. You should upload this as a separate file labeled 'Manuscript'.

If you would like to make changes to your financial disclosure, please include your updated statement in your cover letter. Guidelines for resubmitting your figure files are available below the reviewer comments at the end of this letter.

If applicable, we recommend that you deposit your laboratory protocols in protocols.io to enhance the reproducibility of your results. Protocols.io assigns your protocol its own identifier (DOI) so that it can be cited independently in the future. For instructions see: [https://journals.plos.org/plosone/s/submission-guidelines#loc-laboratory-protocols](https://track.editorialmanager.com/CL0/https:%2F%2Fjournals.plos.org%2Fplosone%2Fs%2Fsubmission-guidelines%23loc-laboratory-protocols/1/010f0198e0125dde-1f16df77-ccf3-4874-a117-679499d1ee7b-000000/2ywEFk6ukBZcRHFU-bbiTLXKXH5jSq2SqKyrUXfOO88=225). Additionally, PLOS ONE offers an option for publishing peer-reviewed Lab Protocol articles, which describe protocols hosted on protocols.io. Read more information on sharing protocols at [https://plos.org/protocols?utm_medium=editorial-email&utm_source=authorletters&utm_campaign=protocols](https://track.editorialmanager.com/CL0/https:%2F%2Fplos.org%2Fprotocols%3Futm_medium=editorial-email%26utm_source=authorletters%26utm_campaign=protocols/1/010f0198e0125dde-1f16df77-ccf3-4874-a117-679499d1ee7b-000000/KPwNYFyhT0svSqNTmyz5tQCn5MANPF7evSQYT29IWB0=225).

We look forward to receiving your revised manuscript.

Kind regards,

Krzysztof Durkalec-Michalski, Ph.D
Academic Editor
PLOS ONE

Journal Requirements:

When submitting your revision, we need you to address these additional requirements.

1. Please ensure that your manuscript meets PLOS ONE's style requirements, including those for file naming. The PLOS ONE style templates can be found at

<https://journals.plos.org/plosone/s/file?id=wjVg/PLOSOne_formatting_sample_main_body.pdf> and

[https://journals.plos.org/plosone/s/file?id=ba62/PLOSOne_formatting_sample_title_authors_affiliations.pdf](https://track.editorialmanager.com/CL0/https:%2F%2Fjournals.plos.org%2Fplosone%2Fs%2Ffile%3Fid=ba62%2FPLOSOne_formatting_sample_title_authors_affiliations.pdf/1/010f0198e0125dde-1f16df77-ccf3-4874-a117-679499d1ee7b-000000/7rej2euOP5iqGg9nf-y14njCFj4X3y8g93wJhmQeQH0=225).

*Thank you for noticing! We have made the appropriate adjustments to the style.*

2. You indicated that you had ethical approval for your study. In your Methods section, please ensure you have also stated whether you obtained consent from parents or guardians of the minors included in the study or whether the research ethics committee or IRB specifically waived the need for their consent.

*Thank you for your comment. In Sweden, parental consent is not required for participants aged 15 years or older. This information has now been added to the Methods section (line 93).*

3. Thank you for stating the following in the Competing Interests section:

“Lisa Söderman and Moa Wolff received an honorarium for an educational webinar by Pharmacosmos.”

We note that one or more of the authors are employed by a commercial company: PharmacosmosK.

a.        Please provide an amended Funding Statement declaring this commercial affiliation, as well as a statement regarding the Role of Funders in your study. If the funding organization did not play a role in the study design, data collection and analysis, decision to publish, or preparation of the manuscript and only provided financial support in the form of authors' salaries and/or research materials, please review your statements relating to the author contributions, and ensure you have specifically and accurately indicated the role(s) that these authors had in your study. You can update author roles in the Author Contributions section of the online submission form.

Please also include the following statement within your amended Funding Statement.

“The funder provided support in the form of salaries for authors [insert relevant initials], but did not have any additional role in the study design, data collection and analysis, decision to publish, or preparation of the manuscript. The specific roles of these authors are articulated in the ‘author contributions’ section.”

If your commercial affiliation did play a role in your study, please state and explain this role within your updated Funding Statement.

*Thank you for the guidance. The Funding Statement has been amended to clarify that there is no employment relationship with the company and that the honorarium was only for the educational webinar, unrelated to the present study. The specific roles of the authors are described in the Author Contributions section.*

b. Please also provide an updated Competing Interests Statement declaring this commercial affiliation along with any other relevant declarations relating to employment, consultancy, patents, products in development, or marketed products, etc.

Within your Competing Interests Statement, please confirm that this commercial affiliation does not alter your adherence to all PLOS ONE policies on sharing data and materials by including the following statement: "This does not alter our adherence to  PLOS ONE policies on sharing data and materials.” (as detailed online in our guide for authors <http://journals.plos.org/plosone/s/competing-interests>) . If this adherence statement is not accurate and  there are restrictions on sharing of data and/or materials, please state these. Please note that we cannot proceed with consideration of your article until this information has been declared.

*Thank you for carefully reviewing the Competing Interests statement. At one single occasion, two of the co-authors gave digital educational lectures aimed at healthcare professionals – one on iron deficiency in adolescents (MW) and the other on menstruation an iron deficiency (LS). For these webinars, they each received a one-time honorarium from Pharmacosmos. However, there is no employment relationship with the company, and these activities were unrelated to the present study. This has now been further clarified in the Competing Interests section.*

*We have added the statement: This does not alter our adherence to PLOS ONE policies on sharing data and materials.*

Please include both an updated Funding Statement and Competing Interests Statement in your cover letter. We will change the online submission form on your behalf.

*Both the updated Funding Statement and the updated Competing Interests Statement have been included in the cover letter, as requested. Thank you for your guidance.*

4. We noted in your submission details that a portion of your manuscript may have been presented or published elsewhere. [The in-depth nutritional and dietary aspects of this study have been presented in “Iron insight: exploring dietary patterns and iron deficiency among teenage girls in Sweden” by Stubbendorf et al. DOI: <https://doi.org/10.1007/s00394-025-03630-z>] Please clarify whether this [conference proceeding or publication] was peer-reviewed and formally published. If this work was previously peer-reviewed and published, in the cover letter please provide the reason that this work does not constitute dual publication and should be included in the current manuscript.

*Thank you for raising this important point. We would like to clarify that there is no dual publication.*

*The previously published article by Stubbendorff et al. focused exclusively on dietary patterns and iron deficiency. In that paper, menstrual data were only included as a confounding variable to adjust the analyses of the relationship between diet and iron deficiency. In contrast, the current manuscript specifically investigates menstrual factors in relation to iron deficiency and provides an in-depth analysis and discussion of these factors, which were not addressed in the previous publication.*

*Therefore, the two manuscripts address distinct research questions and make separate scientific contributions. We have also clarified this distinction in the cover letter to ensure transparency for the editors and reviewers.*

5. We note that you have indicated that there are restrictions to data sharing for this study. For studies involving human research participant data or other sensitive data, we encourage authors to share de-identified or anonymized data. However, when data cannot be publicly shared for ethical reasons, we allow authors to make their data sets available upon request. For information on unacceptable data access restrictions, please see <http://journals.plos.org/plosone/s/data-availability#loc-unacceptable-data-access-restrictions>.

Before we proceed with your manuscript, please address the following prompts:

a) If there are ethical or legal restrictions on sharing a de-identified data set, please explain them in detail (e.g., data contain potentially identifying or sensitive patient information, data are owned by a third-party organization, etc.) and who has imposed them (e.g., a Research Ethics Committee or Institutional Review Board, etc.). Please also provide contact information for a data access committee, ethics committee, or other institutional body to which data requests may be sent.

*Based on the regulations outlined in the approved ethics application, it is not possible to make the data publicly available. Participants were informed that results would only be published at the group level and could not be traced back to individuals. Making the data openly accessible to other researchers could allow identification of individual-level information and would therefore breach compliance with the protocol approved by the Swedish Ethical Review Authority. However, data can be made available upon specific request and arrangements.*

b) If there are no restrictions, please upload the minimal anonymized data set necessary to replicate your study findings to a stable, public repository and provide us with the relevant URLs, DOIs, or accession numbers. Please see <http://www.bmj.com/content/340/bmj.c181.long> for guidelines on how to de-identify and prepare clinical data for publication. For a list of recommended repositories, please see <https://journals.plos.org/plosone/s/recommended-repositories>. You also have the option of uploading the data as Supporting Information files, but we would recommend depositing data directly to a data repository if possible.

Please update your Data Availability statement in the submission form accordingly.

6. In the online submission form you indicate that your data is not available for proprietary reasons and have provided a contact point for accessing this data. Please note that your current contact point is a co-author on this manuscript. According to our Data Policy, the contact point must not be an author on the manuscript and must be an institutional contact, ideally not an individual. Please revise your data statement to a non-author institutional point of contact, such as a data access or ethics committee, and send this to us via return email. Please also include contact information for the third party organization, and please include the full citation of where the data can be found.

*The data underlying our study have now been uploaded to the* ***Swedish National Data Service (SND)*** *repository and are currently under review. The dataset will be available upon reasonable request via* [***request@snd.se***](mailto:request@snd.se) *after publication.*

7. If the reviewer comments include a recommendation to cite specific previously published works, please review and evaluate these publications to determine whether they are relevant and should be cited. There is no requirement to cite these works unless the editor has indicated otherwise.

-

8. Please review your reference list to ensure that it is complete and correct. If you have cited papers that have been retracted, please include the rationale for doing so in the manuscript text, or remove these references and replace them with relevant current references. Any changes to the reference list should be mentioned in the rebuttal letter that accompanies your revised manuscript. If you need to cite a retracted article, indicate the article’s retracted status in the References list and also include a citation and full reference for the retraction notice.

*The reference list has been reviewed.*


[Note: HTML markup is below. Please do not edit.]

Reviewers' comments:

Reviewer's Responses to Questions

**Comments to the Author**

1. Is the manuscript technically sound, and do the data support the conclusions?

The manuscript must describe a technically sound piece of scientific research with data that supports the conclusions. Experiments must have been conducted rigorously, with appropriate controls, replication, and sample sizes. The conclusions must be drawn appropriately based on the data presented.

Reviewer #1: Yes

Reviewer #2: Yes

Reviewer #3: Yes

2. Has the statistical analysis been performed appropriately and rigorously?

Reviewer #1: Yes

Reviewer #2: Yes

Reviewer #3: Yes

3. Have the authors made all data underlying the findings in their manuscript fully available?

The [PLOS Data policy](http://track.editorialmanager.com/CL0/http:%2F%2Fwww.plosone.org%2Fstatic%2Fpolicies.action%23sharing/1/010f0198e0125dde-1f16df77-ccf3-4874-a117-679499d1ee7b-000000/aDmZcqNr02D6VBXsbatM0fWlfpQF7IHAeFttf3G0JY4=225) requires authors to make all data underlying the findings described in their manuscript fully available without restriction, with rare exception (please refer to the Data Availability Statement in the manuscript PDF file). The data should be provided as part of the manuscript or its supporting information, or deposited to a public repository. For example, in addition to summary statistics, the data points behind means, medians and variance measures should be available. If there are restrictions on publicly sharing data—e.g. participant privacy or use of data from a third party—those must be specified.

Reviewer #1: Yes

Reviewer #2: No

Reviewer #3: Yes

4. Is the manuscript presented in an intelligible fashion and written in standard English?

PLOS ONE does not copyedit accepted manuscripts, so the language in submitted articles must be clear, correct, and unambiguous. Any typographical or grammatical errors should be corrected at revision, so please note any specific errors here.

Reviewer #1: Yes

Reviewer #2: Yes

Reviewer #3: Yes

5. Review Comments to the Author

Please use the space provided to explain your answers to the questions above. You may also include additional comments for the author, including concerns about dual publication, research ethics, or publication ethics. (Please upload your review as an attachment if it exceeds 20,000 characters)

Reviewer #1: The authors are commended for undertaking and expertly exploring the prevalence of iron deficiency in adolescents and how it relates to dietary habits and iron deficiency. Using a ferritin cutoff of < 15 to define iron deficiency (WHO recommendation)

As much as it can be inferred that a diet low in meat in addition to HMB can lead to greater iron deficiency, it is helpful to have this data and also apply the SAMANTA questionnaire.

Although there are recommendations to consume less meat for health/ethical reasons, this study raises the questions about how such recommendations can impact a cohort of adolescents at risk for iron deficiency.

Reviewer #2: Whilst the full dataset is not provided, a statement is made that it is accessible with caveats.

Title: Is ‘Iron insight’ needed at the beginning of the title?
*Thank you for your comment. The phrase ‘Iron insight’ has been removed from the title to improve clarity.*

General points:
This paper addresses an important area of health and a widely studied topic.
Good size cohort (n=394), from two schools.
Simple cross sectional study design describing prevalence and then Odds ratios calculated for iron deficiency by group and combined.
Findings not particularly novel but confirmatory of what has been reported elsewhere.
Very well written article with an excellent overview of the literature.

*Thank you!*

The article seems very similar to this article recently published where ferritin levels were reported according to diet types: Stubbendorff, A., Borgström Bolmsjö, B., Bejersten, T. et al. Iron insight: exploring dietary patterns and iron deficiency among teenage girls in Sweden. Eur J Nutr 64, 107 (2025). <https://doi.org/10.1007/s00394-025-03630-z>
The authors need to cite this article and make it clear how the present article is different.
I see that this is explained line 112-114, however, some further explanation would be welcomed, perhaps in the discussion.

*The previously published article by Stubbendorff et al. focused exclusively on dietary patterns and iron deficiency. In that paper, menstrual data were only included as a confounding variable to adjust the analyses of the relationship between diet and iron deficiency. In contrast, the current manuscript specifically investigates menstrual factors in relation to iron deficiency and provides an in-depth analysis and discussion of these factors, which were not addressed in the previous publication. This is now clarified in the Discussion section, line 299.*

Can the authors comment on how representative these school children are of the general population? i.e. the socio-demographic status?

*Thank you for this valuable comment. To better illustrate the representativeness of the cohort, we have added the socio-demographic variable urban/small town/rural to Table 1. This provides an overview of the participants’ backgrounds and clarifies the distribution between those from urban areas, small towns, and rural areas.*
I would recommend supplying the full questionnaires used, including the translations, as supplementary files.
*Thank you for this helpful suggestion. The full questionnaires used, including the translations, have now been added as supplementary information.*

Were samples transported to a laboratory or analysed onsite?

*Thank you for your question. The samples were transported to a laboratory, and this information has now been clarified in the Methods section (line 135).*

Please check the following statement, line 114:
“Those with anemia (Hb <107 g/L as per local reference values) or ferritin >150 μg/L were referred to their primary care center for follow-up.”
Should this be ferritin < 15 ug/L? If it is a threshold for iron overload, please provide an explanation.

*Thank you for this comment and for the opportunity to clarify.
Participants with isolated iron deficiency without anemia were not referred to primary care. Instead, they received written information and recommendations for a three-month course of oral iron supplementation, along with guidance on how to prevent future iron deficiency. They were also provided with contact details for a youth health center (ungdomsmottagning) for further support, including menstrual regulation if needed, and advice to consult a dietitian if they had questions about dietary habits.*

*Participants with anemia (Hb <110 g/L for ages 10–18 or Hb <117 g/L for ages >18, according to local reference values) or with markedly elevated ferritin levels (>300 µg/L, indicating possible iron overload requiring medical assessment) were referred to their primary care center for further evaluation and follow-up (line 145).*

Figure 2 typo: ’stainig’
*Thank you for noticing this typo, it has been corrected.*

Line 160-163 and Fig S1
Please explain how the DAG was constructed - what sources of information were used?
On the DAG figure itself, or caption, please provide more information, e.g. an explanation for the colours of the arrows.

*We used the Dagitty tool. Please find a more elaborated reasoning for what variables we used on line 165.*

*Confounders of the association between HMB and ID, i.e. that covariates that could influence both the risk of HMB and of ID, were selected based on the directed acyclic graph framework and included: BMI and years with menstruation (S1Fig). A higher BMI is associated with heavy menstrual bleeding in young women. Obesity as well as a low BMI is associated with iron deficiency. Participants with fewer years of menstruation are at higher risk of heavy anovulatory bleeding which is more common in the years after menarche. Study participants with younger age at menarche will have had more years with menstruation and are thus at risk of negative iron balance due to loss. The use of iron supplements and dietary restrictions were not considered confounders of the relationship between HMB and ID as per S1Fig.*

*S1 Figure Directed acyclic graph (DAG) for identifying confounders between heavy menstrual bleeding (HMB) and iron deficiency (ID).
Green arrow indicates the “causal” relationship of interest, i.e. between HMB and ID. Red arrows indicate potential biasing paths. Black arrows indicate non-biasing, non-causal paths between variables. For estimating the total effect of HMB on ID, the Dagitty tool (*[*www.dagitty.net*](http://www.dagitty.net)*) suggests adjustment for BMI (body mass index) and years with menstruation.*

Discussion

LIne 288-291. I am somewhat concerned about the suggestion of increasing use of iron replacement therapy, tranexamic acid or hormonal contraceptives in this young population. These come with their own risks and this must be acknowledged here. A decision would need to be made - which one carries a higher health risk? HC use in particular could be counter-productive as it removes the natural health signal of menstruation which has been termed a ‘vital sign’, and carries other health risks. This needs a fuller explanation or should be removed. At the very least non-pharmacological interventions should be considered/attempted first in these young females.

*Thank you for raising this important point. We agree that this is a complex and sensitive issue, and to avoid potential misunderstanding, the passage has been removed from the manuscript.*

Line 350: There is substantial evidence that menstruating females require more iron than males due to menstrual blood losses. Therefore I would suggest that a lower cutoff for females doesn’t lack scientific evidence, in fact it runs counter to the scientific evidence.

*We absolutely agree! The sentence has been rephrased.*

*Interestingly, these laboratory cutoffs are set lower for females than for males, suggesting lower physiological iron requirements in females, which runs counter to scientific evidence (line 363).*

Reviewer #3: Dear Editor, Dear Authors,
Thank you for the opportunity to review the manuscript titled: “Iron insight - Exploring the effect of menstrual loss and dietary iron intake on iron deficiency in teenagers: a cross-sectional study”. The results of this study identify the important problem of iron deficiency of adolescent girls. The manuscript is well written, however I have a few questions and suggestions:

1. The title of the study states, that it “explores dietary iron intake”, however from what I understand from the methods section, this particular manuscript is based on participant self-report of meat restriction diet. There is no information on dietary assessment methods used, therefore we do not know what is the dietary iron intake among the participants. As described in the discussion, the intake of iron itself could be within dietary recommendations, however combining dietary factors, such as consuming vitamin C, phytates, or casein could influence the absorption.

*You are right. We have changed the title to “Exploring the effect of menstrual loss and dietary habits on iron deficiency in teenagers: a cross-sectional study”, as we only look at the intake of meat and use a meat-restricted diet as a proxy for low dietary intake of heme iron, and possibly lower total iron absorption.*

*In depth presentation of the dietary intake of the population is presented in “Iron insight: exploring dietary patterns and iron deficiency among teenage girls in Sweden” by Stubbendorff et al. DOI: 10.1007/s00394-025-03630-z*

2. There is no information on the toxicity of supplemental iron, which I would recommend to add in the discussion. Are there any food products mandatory fortified with iron in Sweden?

*Thank you for this comment. We have now added information in the Discussion about potential risks and side effects of oral iron supplementation (line 321).
When taken according to recommendations, oral iron is generally considered safe, as absorption increases in individuals with iron deficiency and decreases once iron stores are replenished. However, excessive intake or accidental overdose can be associated with toxicity.
Gastrointestinal side effects are also common and may lead to low compliance.
There are currently no mandatory iron-fortified food products in Sweden.*

3. For future consideration, it would be interesting to collect information on physical activity of the participants; regular physical activity seem to reduce risk of menstruation irregularities, including the heavy menstrual bleeding.

*We fully agree, and we have added the lack of information on physical activity to the “weakness” section of the Discussion (line 347).*

*While we were aware of the association between physical activity and iron absorption, we were not familiar with its potential protective effect against heavy menstrual bleeding. We would be very interested in learning more about this and would greatly appreciate it if you could share relevant references.*

6. PLOS authors have the option to publish the peer review history of their article ([what does this mean?](https://track.editorialmanager.com/CL0/https:%2F%2Fjournals.plos.org%2Fplosone%2Fs%2Feditorial-and-peer-review-process%23loc-peer-review-history/1/010f0198e0125dde-1f16df77-ccf3-4874-a117-679499d1ee7b-000000/du8Jk5Pd-UmKjzq8bluwm59oAp9Bo-PZEIz4lo2IHQg=225)). If published, this will include your full peer review and any attached files.


If you choose “no”, your identity will remain anonymous but your review may still be made public.


**Do you want your identity to be public for this peer review?** For information about this choice, including consent withdrawal, please see our [Privacy Policy](https://track.editorialmanager.com/CL0/https:%2F%2Fwww.plos.org%2Fprivacy-policy/1/010f0198e0125dde-1f16df77-ccf3-4874-a117-679499d1ee7b-000000/qjIOPh7c8mZd4i2CuD0n5CTUchcxNnduw_RyQO1EbIA=225).

Reviewer #1: No

Reviewer #2: No

Reviewer #3: No

[NOTE: If reviewer comments were submitted as an attachment file, they will be attached to this email and accessible via the submission site. Please log into your account, locate the manuscript record, and check for the action link "View Attachments". If this link does not appear, there are no attachment files.]

While revising your submission, please upload your figure files to the Preflight Analysis and Conversion Engine (PACE) digital diagnostic tool, [https://pacev2.apexcovantage.com/](https://track.editorialmanager.com/CL0/https:%2F%2Fpacev2.apexcovantage.com%2F/1/010f0198e0125dde-1f16df77-ccf3-4874-a117-679499d1ee7b-000000/yT6DQchi1MQ3dTaJwImGxm-qTxuv-ih2XEGo1T2_GBQ=225). PACE helps ensure that figures meet PLOS requirements. To use PACE, you must first register as a user. Registration is free. Then, login and navigate to the UPLOAD tab, where you will find detailed instructions on how to use the tool. If you encounter any issues or have any questions when using PACE, please email PLOS at [figures@plos.org](mailto:figures@plos.org). Please note that Supporting Information files do not need this step.

*In compliance with data protection regulations, you may request that we remove your personal registration details at any time. [(Remove my information/details)](https://track.editorialmanager.com/CL0/https:%2F%2Fwww.editorialmanager.com%2Fpone%2Flogin.asp%3Fa=r/1/010f0198e0125dde-1f16df77-ccf3-4874-a117-679499d1ee7b-000000/RWfsbUCU9lZDxd3s6_sVB75Jn2zUmB_glecQhRgnLeM=225). Please contact the publication office if you have any questions.*
