# Supplementary material for: Correction: Exploring the effect of menstrual loss and dietary habits on iron deficiency in teenagers: A cross-sectional study
Source: PLoS One. 2026 Feb 17;21(2):e0343133. doi: 10.1371/journal.pone.0343133 (PMC12912581; doi:10.1371/journal.pone.0343133)
Supplement: S1 File — (DOCX) [file pone.0343133.s001.docx]

Exploring the effect of menstrual loss and dietary habits on iron deficiency in teenagers: a cross-sectional study

S1 Questionnaire

| **Swedish** | **English translation** |
| --- | --- |
| Har du fyllt 15 år? | Are you 15 years old or older? |
| Har du fått din första mens? | Have you had your first period? |
| Har du, eller utreds du för, inflammatorisk tarmsjukdom? | Do you have, or are you being investigated for, an inflammatory bowel disease? |
| Har du, eller utreds du för annan kronisk inflammationssjukdom (tex reumatism, SLE, Bechterews sjukdom)? | Do you have, or are you being investigated for, any other chronic inflammatory disease (e.g.rheumatoid arthritis, SLE, Bechterew’s disease)? |
| Har du en pågående antibiotikabehandling mot någon infektion? | Are you currently taking antibiotics for an infection? |
| Är du gravid? | Are you pregnant? |
|  |  |
| Hur bor du?:  I en större stad/samhälle, t ex Malmö/Lund/Landskrona/Trelleborg I en mindre ort på landsbygden, t ex Staffanstorp/Lomma Utanför ort på landsbygden | Where du you live?: In a city (like Malmö/Lund/Landskrona/Trelleborg) In a small rural town (like Staffanstorp/Lomma) In a rural area outside a town |
|  |  |
| Generellt: Hur skulle du säga att din allmänna hälsa är? Dålig Ganska bra Bra Väldigt bra Utmärkt | How would you rate your general health? Bad Pretty good Good Very good Excellent |
|  |  |
| Röker du? (cigaretter/e-cigaretter) Nej Ja, dagligen Ja, sporadiskt/feströker | Do you smoke? No Yes, daily Yes, occasionally |
| Snusar du? Nej Ja, dagligen Ja, sporadiskt/feströker | Do you use snuff? No Yes, daily Yes, occasionally |
| Tar du järntillskott dagligen eller flera gånger per vecka (utöver eventuell multivitamintablett)? Ja Nej Vet ej | Do you take an iron supplement daily or several times per week (in addition to any multivitamin tablet)? Yes No Don't know |
| Hur gammal var du när du fick mens första gången? | How old were you when you had your first period? |
| Använder du något hormonberoende preventivmedel? Nej P-piller Minipiller P-stav Hormonspiral Annat | Do you use any hormonal contraceptive? No Combined pill Minipill (Progestin-only) Contraceptive implant Hormonal IUS (intrauterine system) Other |
| **SAMANTA** |  |
| Blöder du mer än 7 dagar per månad? | Do you experience menstrual bleeding for more than seven days per month? |
| Har du 3 eller fler dagar med riklig blödning i samband med menstruation? | Do you experience three or more days of heavier menstrual bleeding during your menstrual period? |
| I allmänhet, upplever du din mens som speciellt obehaglig på grund av att den är så riklig? | In general, does menstruation bother you due to its abundance? |
| Under de dagar du blöder som mest: Får du blodfläckar på kläder/lakan på natten, eller skulle du få det om du inte använde dubbelt skydd eller bytte mensskydd under natten? | During any of these heavier menstrual bleeding days, do you spot your clothes at night, or would you spot them if you did not use double protection/did not change your clothes during the night? |
| Under de dagar du blöder som mest: Oroar du dig för att få mensfläckar på stolsits, soffa etc.? | During these heavier menstrual days, are you worried about staining the chair, sofa, etc.? |
| I allmänhet, undviker du (om möjligt) vissa aktiviteter, resor eller fritidsplaner under de dagar du blöder som mest eftersom du måste byta tampong eller binda ofta? | In general, during these heavier menstrual bleeding days, do you avoid, as far as possible, some activities, trips, or leisure-time plans because you frequently need to change your tampon or sanitary towel? |
